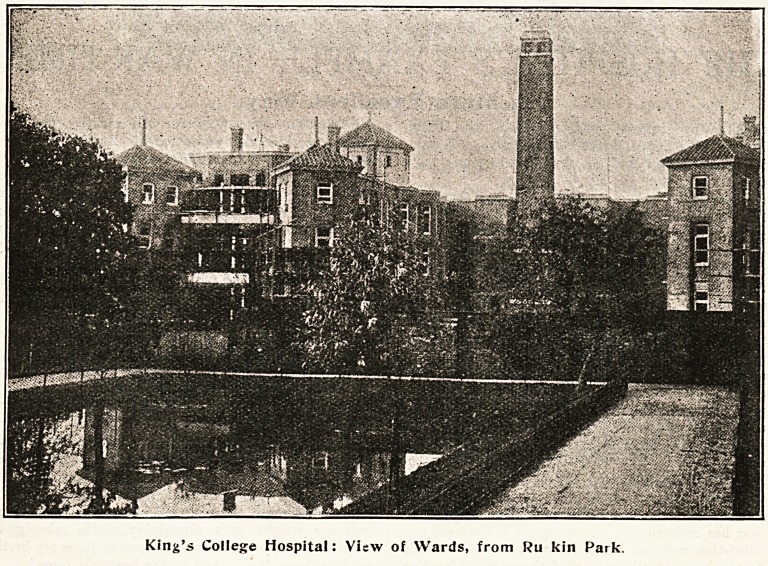# The Hospital's Example in War-Time: A More Excellent Way

**Published:** 1915-12-18

**Authors:** 


AMBULANCE DUTY WITH WAR TRAIN FROM THE FRONT. 3rd L.G.H.

The Muster of the Station Party, 1 a.m. By Private W. R. S. Stott.

### THE HOSPITAL'S EXAMPLE IN WAR-TIME.

### A More Excellent Way.

THE following sermon was preached by Bishop Frodsham in All Saints' Church, Cheltenham, on November 26 from the text "And yet shew I unto you a more excellent way" (1 Cor. xii. 31) :—

To-day we are filled with pride at the charitable activities of English women and girls. Gloucestershire stands in the front rank of Red Cross work. There is not one man in this church who does not feel tender admiration of the spirit of our women-folk to-day. It will not be out of place, therefore, to remind you that it was a Christian lady called Fabiola who did more than any other single individual in the erection of the first hospital in Rome. Moreover, during the Crusades, when Gloucester was one of the three centres of national life, the wives of the Crusaders were noted for being the tender nurses and devoted doctors of dying strangers—strangers who under the cruel laws of those days had no rights. There was an old saying then, coming from Roman times, to the effect that "a man is a wolf to a man he does not know." The white cross was the badge of the Crusaders. They thought they did God service by killing infidels. The blood-red cross is the mark of the women who tend with tireless hands those whom this cruel war has maimed.

No Christian man can avoid feeling humiliated at the fact of this war. If all who called themselves Christian had been more Christlike there would have been no call for Englishmen to fight for the sanctity of national treaties nor for the protection of English women and English homes. Alas that the war is necessary! We men *must* see it through. But, and this is my point, the women, moved by a self-sacrifice equal to the men, have shown us how the rule of Christ in the world may be made a more living thing than it was before the war. If the hospitals have been from the first the glory of Christianity, then the new Red Cross activity can become a means whereby the Prince of Peace will bless again this blood-stained world and make it more truly Christian.

The movement of the women has been supremely patriotic. God forbid that I should say one word against a patriotism that makes men and women unselfish, brave, and kind. But will it be less patriotic, less unselfish, less brave, less kind to do all that is being done more definitely according to the mind of Christ? If there is any bright beam of light thrown across the present situation it comes from the living spirit of gentleness and love towards the sick and wounded. None can doubt that hospital work is a true fruit of Christ's teaching. But Christ never spoke of hospitals. He laid no extraordinary weight upon almsgiving. He simply said, "Inasmuch as ye do it unto the least of these my brethren ye do it unto Me." And so the hospitals came!

This is not all. In the light of the Cross not only charity attains a new significance but all suffering is taking a truer interpretation. In a Red Cross hospital in Gloucester a fortnight ago some wounded soldiers were talking among themselves about all those things that have happened in Belgium and France—things which they themselves had seen. One of their number wrote to me this little note, "Please, Bishop, will you tell us straight why God lets innocent people suffer? Does God really care about it? We want to know." How could I fail to respond to this request? And yet can you not see how hard it is for any honest man to answer such a question to his own satisfaction, let alone to the satisfaction of men who had seen and borne such suffering themselves? Speaking to the men two nights later I said I believed God cared because of the price He had paid Himself to win men back from sin. Jesus upon the Cross is not only a spectacle of a poor, good man done to death with selfish, fiendish cruelty. He displays there the mind of God and the method of God. Jesus willingly offered Himself to death to bring men back from sin by His love. "And I, if I be lifted up," He said, "will draw all men unto Me." I tell you this attraction of a loving Christ is true now. The other day I sent a wounded soldier into the operating-room holding tight in his hand a little crucifix I had given him. He smiled as I left him. He *knew* God cared for him because of that outward sign of God's inestimable love.

I don't pretend that I can understand it all. But does not the mystery of the Cross explain the mystery of human suffering to-day? Why, the wounded soldiers themselves, worn, torn, and mangled, so simple, so natural, so brave, so infinitely pathetic, have one appeal not unlike that of Christ crucified Himself. They are suffering willingly, and their suffering calls forth our love and tenderness. It is love alone that can heal this sick world. It is love that is curing us of selfishness and frivolity. It is love that is making us anxious to follow Him Who went about doing good. Can't you feel it as a real thing? Can't you see that it is Christ in the person of His wounded soldiers Who is calling you to Him and to His service? He is reaching down to us to-day also through this welter of innocent suffering to save the world from selfishness and sin. We cannot understand it all yet, we cannot explain it, but we are not as men and women without hope. Afterwards, the wounded soldier who wrote asking if God cared sent me this verbal message, "Tell the Bishop we chaps are satisfied." Isn't that just like a soldier's message? So tantalising in its brevity. But it is a voice of encouragement to faith not to be despised in such times as these.

I have spoken much about Red Cross work because I consider it to be the most remarkable and important development of Christian activities during the present war. It would be a hideous mistake to allow it to be thought that the general hospital work does not matter, or, at least, that it is of secondary importance. It is therefore with not a little relief I read in The Hospital that the Hospital Sunday collections so far from decreasing since the commencement of the war have increased everywhere. Churches where the springs of Christian charity were stagnant, even decreasing in their flow, have shown new life and abundant development. The Hospital is right in saying that no single fact is more encouraging to those who are concerned with the future of the voluntary system. But I for one do not hesitate in claiming all this for Christ Himself. Christ is being revealed to this age as to no other age. The darkness and din of strife cannot disguise the fact that Christ seems more and more desirable, more and more necessary to our race. Shame on us Christian brethren if we allow Him to suffer in the persons of the sick and suffering in our midst.

**King's College Hospital: View of Wards, from Ru kin Park.**

## Figures and Tables

**Figure f1:**
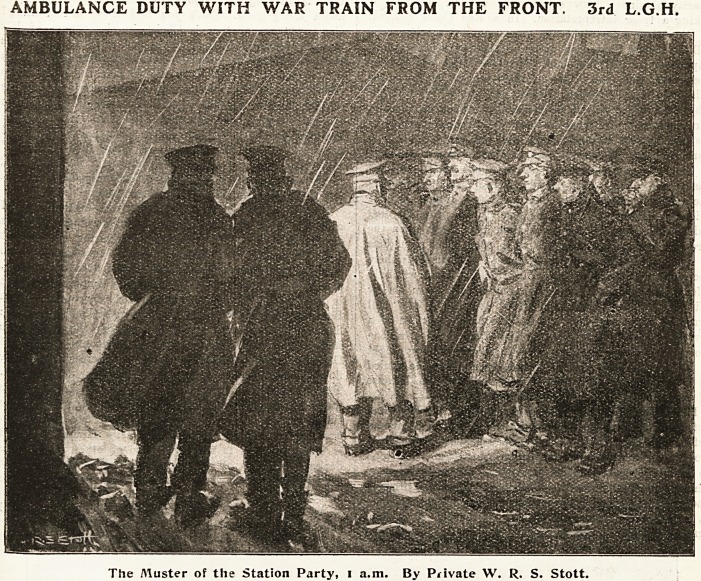


**Figure f2:**